# Hypnotizability, Hypnosis and Prepulse Inhibition of the Startle Reflex in Healthy Women: An ERP Analysis

**DOI:** 10.1371/journal.pone.0079605

**Published:** 2013-11-22

**Authors:** Vilfredo De Pascalis, Emanuela Russo

**Affiliations:** Department of Psychology “La Sapienza” University of Rome, Rome, Italy; Osaka University Graduate School of Medicine, Japan

## Abstract

A working model of the neurophysiology of hypnosis suggests that highly hypnotizable individuals (HHs) have more effective frontal attentional systems implementing control, monitoring performance, and inhibiting unwanted stimuli from conscious awareness, than low hypnotizable individuals (LHs). Recent studies, using prepulse inhibition (PPI) of the auditory startle reflex (ASR), suggest that HHs, in the waking condition, may show reduced sensory gating although they may selectively attend and disattend different stimuli. Using a within subject design and a strict subject selection procedure, in waking and hypnosis conditions we tested whether HHs compared to LHs showed a significantly lower inhibition of the ASR and startle-related brain activity in both time and intracerebral source localization domains. HHs, as compared to LH participants, exhibited (a) longer latency of the eyeblink startle reflex, (b) reduced N100 responses to startle stimuli, and (c) higher PPI of eyeblink startle and of the P200 and P300 waves. Hypnosis yielded smaller N100 waves to startle stimuli and greater PPI of this component than in the waking condition. sLORETA analysis revealed that, for the N100 (107 msec) elicited during startle trials, HHs had a smaller activation in the left parietal lobe (BA2/40) than LHs. Auditory pulses of pulse-with prepulse trials in HHs yielded less activity of the P300 (280 msec) wave than LHs, in the cingulate and posterior cingulate gyrus (BA23/31). The present results, on the whole, are in the opposite direction to PPI findings on hypnotizability previously reported in the literature. These results provide support to the neuropsychophysiological model that HHs have more effective sensory integration and gating (or filtering) of irrelevant stimuli than LHs.

## Introduction

Hypnotizability, generally defined as the ability to enter a hypnotic state, is a complex behavioural phenomenon with biological, cognitive and social components. Hypnosis requires an individual to mainly attend the hypnotist's voice while disattending/ignoring distracting thoughts and stimuli. Accordingly, there is a broad consensus in considering hypnotic susceptibility as an individual characteristic closely related to the ability to focus and sustain attention on relevant stimuli and to shut-off irrelevant ones [1], [2]. A number of studies have demonstrated a more effective frontal attentional control system in high hypnotizable (HH) individuals than low hypnotizable (LH) individuals [3]–[7]. The HHs, as compared to LH individuals, typically demonstrate faster reaction times during complex decision-making tasks [8]–[10] and shorter peak latencies for auditory, visual, and somatosensory components of the event-related potential (ERP) [11]–[13]. While the neurobiological substrates of hypnosis have not been resolved, recent findings indicate that the attentional skills involved in hypnotizability may correlate with central dopaminergic activity [14], [15].

The auditory startle response (ASR) is a ubiquitous, cross-species defensive reflex consisting of a rapid sequential contraction of the orbicularis oculi muscle, evoked by intense acoustic stimuli, with the likely purpose to protect the body from a sudden attack. Although ASR is a tool to study innate fear responses, it is not considered a specific component of the fear reaction per se, but rather a reaction to novel and potentially harmful stimuli [16]. A large body of research has shown that the amplitude of the eyeblink portion of the response can be modulated by attentional, cognitive, and emotional states [17]–[19] engaging frontal brain regions, which are also involved in the secondary emotional responses to the defensive startle [20]. Very recently, research has demonstrated that attentional control using Buddhist meditation reduces ASRs [21]. Moreover, it has been demonstrated that Buddhist meditation-concentration practice leads to altered states similar to those in hypnosis, both phenomenologically and neurologically [22].

Prepulse inhibition (PPI) of the startle response is a technique for assessing sensorimotor gating [23]. A subject hears via earphones a “prepulse,” which is a brief noise at a decibel level that would not ordinarily arouse a startle reaction such as blinking. This stimulus is then followed by a second, stronger stimulus, the pulse, which without the prior prepulse would be expected to cause a startle response (blinking). However, when the prepulse is presented immediately before the pulse, with the interval between the two stimuli too brief to be consciously perceived, the test subject will be less likely to blink. That is to say, even though the subject is unaware of the prepulse, it serves to inhibit the subsequent startle reaction upon hearing the pulse. The degree to which the prepulse inhibits the blink response following the pulse is the PPI and is assumed to reflect the efficiency of the sensorimotor gating system [24]. As with hypnosis, increased dopaminergic tone is suggested to be associated with reduced PPI [25].

The blink response is not alone in being subject to PPI. For example, several middle- and late-latency components of the auditory evoked potential (P30, P50, N100, P200, P300) are differentially susceptible to inhibition by prepulses and used as gating indexes [26]–[32]. Although blinks are muscular and ERPs are neural, research has demonstrated that N100 and P200 may reflect activity of both specific and nonspecific systems [29], [30], [33]. A pronounced inhibition of N100 was found when a weak prepulse was used 100 ms before the pulse to which subjects were attending selectively [34]. Research found reduced N100 and P200 gating in schizophrenia [34], [35] and in cocaine abusers [36].

An inverse correlation between PPI and hypnotizability has been reported [37], a finding which is in line with previous reports suggesting a significant association of hypnotizability with attentional skills and dopaminergic activity [14], [15], [38], [39]. Recently, Levin and colleagues replicated this inverse correlation between hypnotizability and PPI [40], and suggested a dysfunctional pattern of sensorimotor gating in HHs. This conclusion seems to contradict Horton and colleagues [41] findings that HHs, who demonstrated more effective attentional and inhibitory capabilities, had a significantly larger rostrum, a corpus callosum area involved in the allocation of attention and transfer of information between prefrontal cortices, than LHs. According to this view, the mechanisms of sensorimotor gating, which play a crucial role in attentional processes and sensory input, should be more efficient in HHs compared to LHs [3], [4], [41]. In agreement with this sight appears the negative correlation between hypnotizability and blink rate recently reported by Lichtenberg and colleagues [42]. The authors concluded that their observations did not provide evidence for a role of dopamine in determining hypnotizability and suggested that other mechanisms come into play in the neurophysiological underpinnings of hypnosis. In sum, although a contribution of dopaminergic mechanisms to the physiology of the PPI phenomenon has been demonstrated [43], [44], how individual differences in hypnotic susceptibility can influence PPI and ASR mechanisms still remains unclear. Thus, to disentangle these apparent conflicting findings we carried out the present study to validate Lichtenberg and colleagues' findings [37], [40] of reduced PPIs and ASRs in HHs during waking. Mainly we want to extend previous hypnotizability/PPI findings to auditory N100, P200, and P300 gating by employing a PPI paradigm at three lead intervals (30, 60, and 120 msec). Consistent with previous reports [26], [30], [45], we expected to find a gating enhancement of N100, P200, and P300 waves as prepulse-to-pulse interval increases. Since there are no studies comparing ASR and PPI responses in HH and LH individuals during hypnosis, aim of the present investigation was also to test whether the negative association between PPI and hypnotizability reported by Lichtenberg and colleagues [37] is still valid in a state of eyes-open hypnosis in the absence of goal directed activity. We expect that hypnosis, in HHs, should reduce the magnitude of ASR and enhance PPI, and that these effects should be reflected in N100, P200 and P300 components of the ERPs.

Functional magnetic resonance neuroimaging (fMRI) studies in humans have outlined the importance of a prefrontal-striatal-pallido-thalamic circuitry as the modulator of PPI [46], [47]. Source localization studies have reported multiple generators for the N100 and P200 waves located in non-specific regions, such as the cingulate cortex in the limbic lobe, and other regions in the frontal lobe [44], [48], [49]. Studies computing tomographic, functional brain images of the cortical distribution of EEG activity (low resolution electromagnetic tomography, LORETA) [50] have shown that this method is able to distinguish individuals differing in hypnotizability level [51], and between hypnosis and waking conditions [52], [53], [54]. Thus, we used the standardized version of the LORETA system (sLORETA) to substantiate the role of the main cortical substrates sensitive to individual differences in hypnotizability and hypnosis in terms of ASR and PPI, reporting related ERP components (N100, P200, and P300), combined with their source localization analysis. Very recently, using sLORETA source localization method we detected the distributed sources of N100 and P200 ERP components to auditory startle [45] respectively in the right frontal lobe (dorsolateral prefrontal cortex, BA8 and BA9) and in the parietal lobe (right and left precuneus, BA7). On the basis of the above mentioned reports, we expected to find that auditory startle activates prefrontal, cingulate, insular, and precuneus regions of the cortex and that frontal and parietal regions should be sensitive to individual differences in hypnotizability, and differences between waking and eyes-open hypnosis conditions.

## Methods

### Ethics statement

The research was conducted according to the ethical standards of the American Psychological Association (APA) and approved by the Ethics Committee of the Department of Psychology, La Sapienza University of Rome, Italy (2010). Participants were seen individually in the lab and, upon arrival, were informed about the nature of the study. All of them gave their written informed consent for participation in the study.

### Participants, hypnotizability, personality, PPI, and ERP data

Sixty-one right-handed women volunteers were recruited through university courses by advertisements. Handedness was measured by the Italian version of the Edinburgh Inventory Questionnaire [55]. Only physically healthy participants were included. Inclusion criterion demanded the absence of any lifetime history of hearing problems, significant psychiatric or neurologic disease, drug abuse, head trauma or loss of consciousness, treatment with antipsychotic medication, substance abuse or dependence use of amphetamine or cocaine (excluding caffeine and nicotine). This information was obtained using a self-report questionnaire. The subjects were asked to refrain from smoking or drinking coffee for at least three hours before the EEG recording. One participant was excluded from the study since she reported a head trauma. Thus, sixty women participated in the project. Mean age was 24.8, SD = 3.9 yr (range 19–35 yr). Twenty-one HH (M = 9.6, SD = 0.8, N = 21) and 20 LH subjects (M = 2.7, SD = 1.4, N = 20) were first selected using the Italian version of the Stanford Hypnotic Susceptibility Scale, Form C (M = 6.3, SD = 3.1, N = 60) [56], [57]. Participants were designated as being HH or LH subjects when they respectively scored ≥9 and ≤4 on the SHSS:C, according to the Italian norms of this scale [56]. These selected subjects underwent PPI testing. Six HH participants and 5 LH participants failed the PPI testing (details below), thus only 15 HHs (M = 9.5, SD = 0.5) and 15 LH subjects (M = 2.5, SD = 1.5) were considered for statistical analyses. Of these participants, 7 were unhabitual smokers, i.e., they smoked no more than 15 cigarettes per day. The subjects were all women since there are reports indicating that women are significantly more susceptible to hypnosis than men [58], [59], although more recent research has demonstrated that gender difference seems to be rather small even when found [60] and that gender is a moderating variable in the relationship between EEG asymmetry and hypnotizability [61], [62]. Moreover, in terms of gender differences on ASRs, there are findings indicating larger ASRs and weaker PPI in women compared to men [19], [63]. Considering that previous research has demonstrated that PPI is reduced in luteal women compared to follicular women [64], participants who were in a menstrual period were invited for the EEG recordings between the 5th and 11th day after the onset of menses. Since there is no clear demonstration that hypnotic susceptibility changes across menstrual cycle phase, PPI and hypnotic susceptibility levels were evaluated in late noon of the same day.

The following personality measures were obtained: (1) Fear Survey Schedule (FSS) [65], [66]; (2) State and trait anxiety, measured using the State-Trait Anxiety Inventory (STAI-Y1 and STAI-Y2) [67].

PPI was obtained as described in [68], [69]. Testing required approximately 19 minutes, consisting of two trial blocks that included 60 startle stimuli. Block 1 (BL1) and Block 2 (BL2) included 12 PA trials each plus 36 prepulse-pulse trials (12 for each of the three designated prepulse-pulse intervals 30, 60 and 120 ms), and 12 no-stimulus trials (0 dB above background) presented in pseudorandom order. A relaxing period of about 1 min between blocks was given to each participant to avoid boredom during the EEG recording. Startle response was measured as the mean of all responses to a 115-decibel stimulus pulse as reported in [68]. Startle blink amplitudes were then logarithm transformed to reduce anomalies in the kurtosis and skewness usually observed in the distribution of the startle amplitude measure. Prepulse inhibition was computed as the percentage of reduction in amplitude of blink response to a pulse when it was preceded by a prepulse, using the formula: PPI = (PA−PP)/PA×100, where PA indicates amplitude of blink response in response to single pulses, while PP is amplitude of blink response to pulse-with prepulse (PP) trials. We examined blink response to pulses occurring at intervals of 30, 60, and 120 ms after the prepulse (PPI-30, PPI-60, and PPI-120, respectively). Subjects without an adequate baseline blink response of at least 192.5 µV to the first six pulses (Block 1) were excluded from the study, according to the criteria of Braff and collaborators [68].

Acoustic stimuli were delivered during two counterbalanced eyes-open conditions, waking and eyes-open hypnosis, while participants were invited to look straight ahead to a circular fixation point presented in the center of a 15-inch monitor. Following the administration of hypnotic induction (SHSS:C), an eyes-open hypnosis condition was obtained by suggestion.

EEG data were recorded from 22 scalp sites (Fp1, Fp2, F3, F4, T3, T4, FC3, FC4, C3, C4, CP3, CP4, P3, P4, O1, O2, Fz, FCz, Cz, CPz, Pz, Oz) using a pure-tin electrode electrocap referenced to digitally linked ears [(A1+A2)/2]. Procedure for electrophysiological recordings, ocular correction [70] epoching and artifacts rejection [71] were applied. The N100 (M±SE = 107±1.2 msec), P200 (185±2.5 msec), and P300 (280±5.6 msec) waves were detected.

In the next step, sLORETA was used for further analysis of ERP responses. This method enables the spatial identification and analysis of brain cortical activity via conventional EEG recordings [50], [72]–[74] and has proved useful for the analysis of different time segments of ERPs [75]–[77]. sLORETA computes current density (i.e., the amount of electrical current flowing through a solid) without assuming any number of active sources [78]. The sLORETA solution space (i.e., the locations in which sources can be found) is composed of 6239 cubic elements (“voxels”, 5 mm^3^) and is limited to cortical gray matter and hippocampi, as defined by a digitized MRI available from the Montreal Neurologic Institute (MNI; Montreal, Quebec, Canada) [79]–[84]. sLORETA source localization was calculated using coordinates of the 22 electrode positions for every subject at the mean N100, P200, and P300 peaks (averaged across subjects and both hemispheres) for the PA trials (Block-1). For wave source reconstructions and to detect differences in source activity, the subtractions of ERP traces between LH and HH participants, were assessed using sLORETA within time intervals of 80–130 msec, 140–230 msec, 250–320 msec respectively, for the N100, P200, and P300 waves. It is important to note that this localization is not a complete listing of all significantly different cortical areas, but a listing of the local maxima of these differences.

Electrophysiological data and signals are stored in our lab archive and are available upon request.

Full description of hypnosis procedure, personality measures and EEG processings are available on request at the following e-mail address: vilfredo.depascalis@uniroma1.it

### Statistical analyses

A separate ANOVA (glm procedure, SAS 9.2) was performed for EMG startle of PA trials and for each peak amplitude measure of the N100, P200, and P300 waves. This analysis was focused on 3 head levels of the lateral dimension (left, midline, and right), each including 5 recording sites (i.e, frontal, fronto-central, central, centro-parietal, and parietal sites; for left side: F3, FC3, C3, CP3, and P3; for central side: Fz, FCz, Cz, CPz, and Pz; and for right side: F4, FC4, C4, CP4, and P4). The ANOVA design was the following: 2 Hypnotizability (high, low)×2 Condition (waking, hypnosis)×3 Head level (left, mid, right)×5 Recording site (frontal, fronto-central, central, centro-parietal, and parietal).

Similar ANOVAs were also performed for PP trials on EMG, N100, P200, and P300 peak amplitudes to evaluate effects of Hypnotizability, Condition, PPI (PPI-30, PPI-60, PPI-120 msec), Head level, and Recording site. Similar analyses were used. To prevent the risk of type-I errors, as may happen using repeated measures analysis if the sphericity assumption has been violated [85], the Huynh-Feldt epsilon correction of significance levels was applied when necessary. An alpha of .05 was used for all post-hoc comparisons [86]. Post-hoc comparisons of the means were performed by using a t-test procedure with α = .05 and the Bonferroni correction was used to control for Type I error inflation [87] when necessary. *P*-value after the correction is reported.

## Results

### Hypnotizability, anxiety, and fear

There were no statistically significant differences between HH and LH groups with respect to state anxiety, trait anxiety, and fear. Means and t-test values for these measures are reported in Table 1.

**Table 1:** Mean, standard deviation (STD) and t values of high (HH) vs. low (LH) hypnotizable subjects for the measures of hypnotizability (SHSS:C and HIP), state anxiety pre- and post- experimental session (STAI-Y1_pre, STAI-Y1_post), trait anxiety (STAI-Y2) and fear.

HH Subjects (N = 15)			LH Subjects (N = 15)		
Variable	Mean	STD	Mean	STD	t value
**SHSSC**	9.5	0.5	2.5	1.6	16.41†
**HIP**	8.1	0.8	2.7	1.1	15.52†
**STAI-Y1_pre**	36.5	11.0	33.2	6.4	1.04
**STAI-Y1_post**	37.0	14.2	33.2	8.4	0.93
**STAI-Y2**	48.1	11.9	44.2	7.9	1.05
**FSS**	141.0	47.8	132.9	46.8	0.47

† p<.0001.

### EMG amplitude and latency to PA startle

EMG-startle amplitude to PA failed to evidence significant differences between hypnotizability levels, or between waking and hypnosis conditions, or for the interaction of Hypnotizability with Condition [all Fs<1]. However, EMG peak latency to PA yielded a significant main effect of Hypnotizability [F(1, 28) = 5.28, p = .029] indicating shorter EMG peak latencies in LH as compared to HHs (54.4±1.9 msec vs. 58.9±1.5 msec, respectively). No other effects were found to be significant.

### N100, P200, and P300 peak amplitudes to PA startle

**Figure 1:**
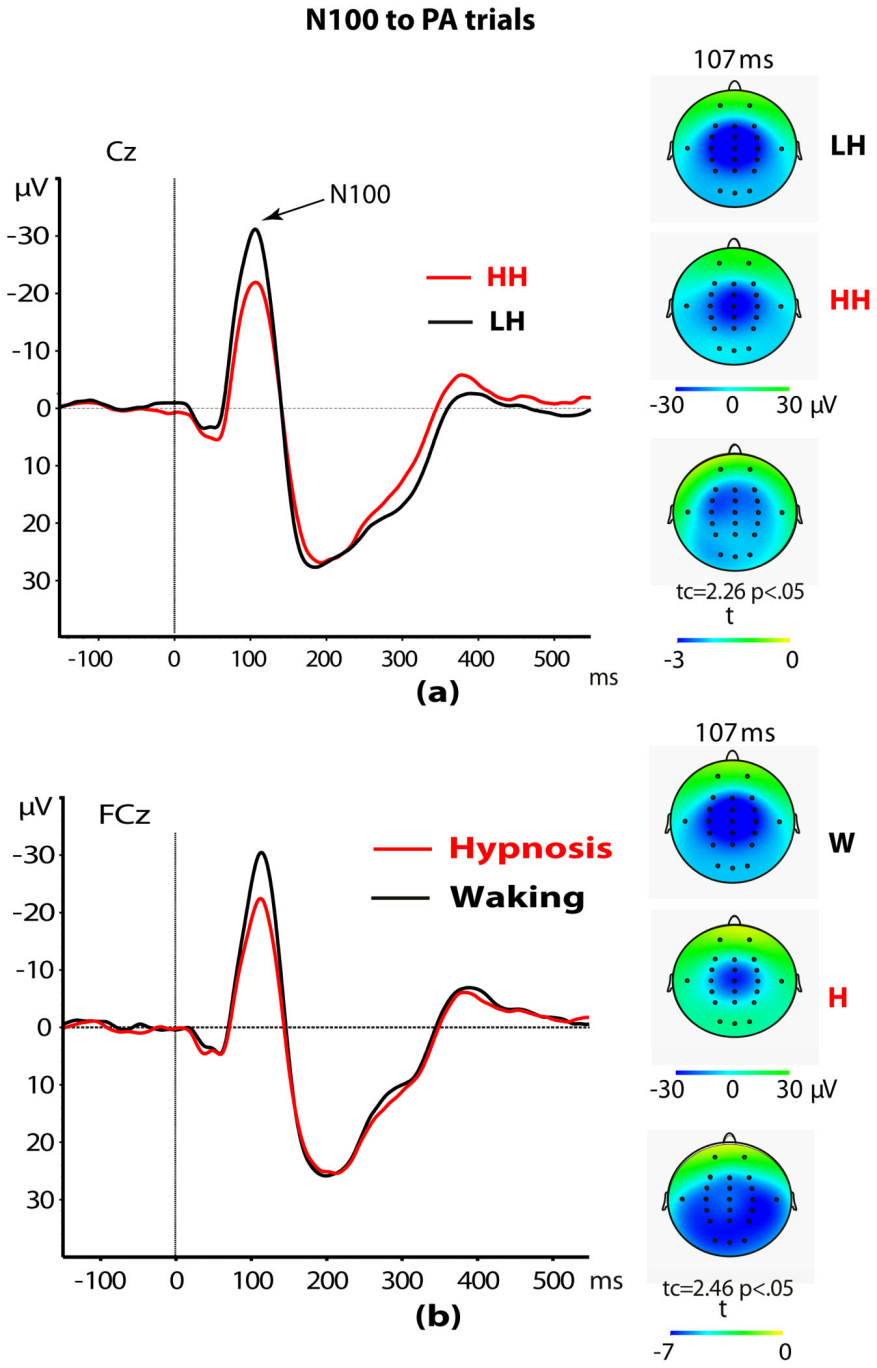
Prepulse inhibition of eyeblink startle response across 30, 60, and 120(_*_ p<.05).

Some effects of interest emerged from the four-way split-plot ANOVA performed on N100 amplitudes obtained for PA stimuli. A main effect of Hypnotizability was detected [F(1,28) = 5.12, p = .031]. This effect disclosed higher N100 amplitudes in LH participants as compared to HH ones (−22.3±1.8 µV vs. −16.8±1.5 µV; see Figure 1a). Further, N100 wave in hypnosis was smaller than that detected in the waking condition [F(1,28) = 5.92, p = .022; see Figure 1b]. In addition, both Head level and Recording site, and their interaction, were all significant [F(2,56) = 39.08, p<.0001; F(4,112) = 13.00, p<.0001; F(8,224) = 5.70, p<.0001, respectively]. The first effect indicated that N100 wave was larger over mid head level as compared with left and right levels (−23.7±1.6 µV vs. −17.1±1.1 µV and −17.8±1.3 µV, t(29) = −7.8, and −5.9, p<.0001, respectively). The second effect showed that across frontocentral and central sites there were larger N100 waves than frontal, centroparietal and parietal sites (−23.3±1.6 µV and −22.1±1.4 µV vs. −19.1±1.8 µV, −19.4±1.4 µV and −13.9±1.3 µV, respectively; for these comparisons all pairwise *t* tests ranged from 2.7 to 7.8 and were significant, p<.01). The interaction effect indicated that N100 wave was higher over both FCz and Cz sites, compared to the other recording sites with the exclusion of CPz site (FCz: −28.4±2.1 and Cz: −27.6±2.0 vs. Fz: −22.5±2.2, CPz: −24.9±1.9, Pz: −15.4±1.5, F3: −16.8±1.9, FC3: −19.7±1.6, C3: −19.1±1.1, CP3: −16.8±1.2, P3: −13.0±1.2, F4: −18.1±2.4, FC4: −21.6±1.7, C4: −19.7±1.3, CP4: −16.5±1.2, P4: −13.3±1.4; with exception of CPz, all post-hoc pairwise *t* tests were significant, p<.01). No other interaction reached significance.

The ANOVA performed on P200 amplitudes to PA stimuli yielded significant effects for both Head level, Recording site and the interaction between these two factors [F(2,56) = 90.64, p<.0001; F(4,112) = 9.45, p = .001; F(8,224) = 11.49, p<.0001, respectively]. These three effects, taken together, indicated that the FCz, Cz, and CPz sites, compared to the other recording sites, had significantly more pronounced P200 waves (all post-hoc pairwise *t* tests were highly significant after Bonferroni correction, p<.01). No other significant effects were observed for this ERP component.

The ANOVA on P300 amplitudes to PA stimuli did not yield significant effects involving Hypnotizability and Condition. However, these scores were sensitive to Recording site [F(4,112) = 18.75, p<.0001] and indicated that there was a progressive increase of the P300 amplitude from anterior to posterior sites. Mean scores across frontal, frontocentral, central, centroparietal, and parietal sites (16.64±1.23, 19.48±1.31, 21.68±1.37, 22.98±1.37, 23.40±1.27, respectively) were compared using pairwise t tests that were all significant (p<.01 after Bonferroni correction), with the exception of the centroparietal vs. parietal comparison that did not reach the significance level (t(29) = 1.37, p = 0.176). No other interactions reached significance for P300.

### Prepulse Inhibition of the eyeblink reflex, N100, P200, and P300 peak amplitudes

**Figure 2:**
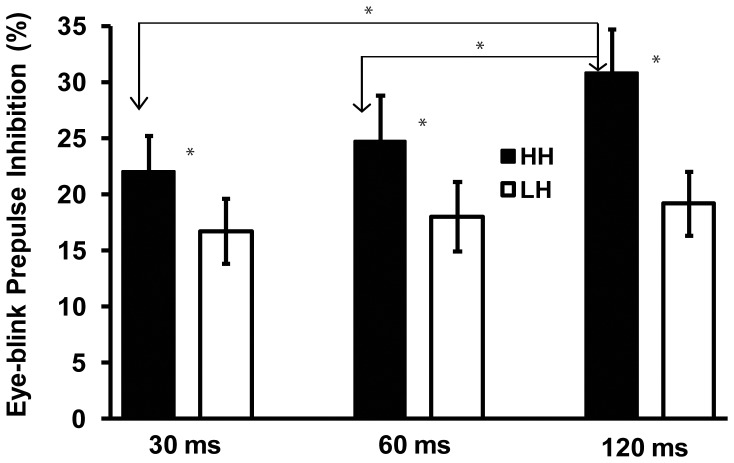
ERP response at Cz to pulse-alone (PA) trials and scalp topography of N100 wave at 107 msec in high (HH) and low (LH) hypnotizable participants (panel a). ERP responses at FCz to PA startle and scalp topography of N100 wave at 107(panel b). t-Test maps are shown in the upper and lower right panels.

A three-way ANOVA on PPI scores of the startle blink evidenced a significant effect of Hypnotizability [F(1, 28) = 4.24, p<.05]. This effect showed higher PPI scores in HHs as compared to LHs (25.8%±2.9 vs. 18.1±2.3%, respectively for M±SE). The interaction between Hypnotizability and Trial was also significant [ F(2, 56) = 4.24, p<.05]. This interaction disclosed a greater PPI score at 120 msec interval as compared to 60 and 30 msec intervals in HHs [t(14) = 2.55, p = .017 and t(14) = 2.72, p = .015], while no significant differences across trials were observed in LH participants (see Figure 2). No other interaction effects reached significance.

The ANOVA on PPI scores of the N100 amplitude evidenced a main effect for Condition [F(1,28) = 5.94, p = .021] that indicated a greater PPI across all lead intervals during hypnosis as compared to the waking condition (71.1±5.2 vs. 50.9±5.4, respectively). This analysis did not evidence other significant effects, although the effect of PPI factor was near significance [F(2,56) = 2.95, p = .06]. Post-hoc pairwise *t* tests between PPIs disclosed a significantly smaller inhibition of the N100 wave for PPI-30 msec as compared to PPI-60 and PPI-120 [t(29) = −3.12, p<0.01, and t(29) = −3.87, p = 0.001; 32.9±6.9%, 71.7±5.3%, and 78.4±4.2%, respectively for mean scores of PPI-30, PPI-60, and PPI-120]. No significant differences were detected between PPI-60 vs. PPI-120 msec (t<1).

**Figure 3:**
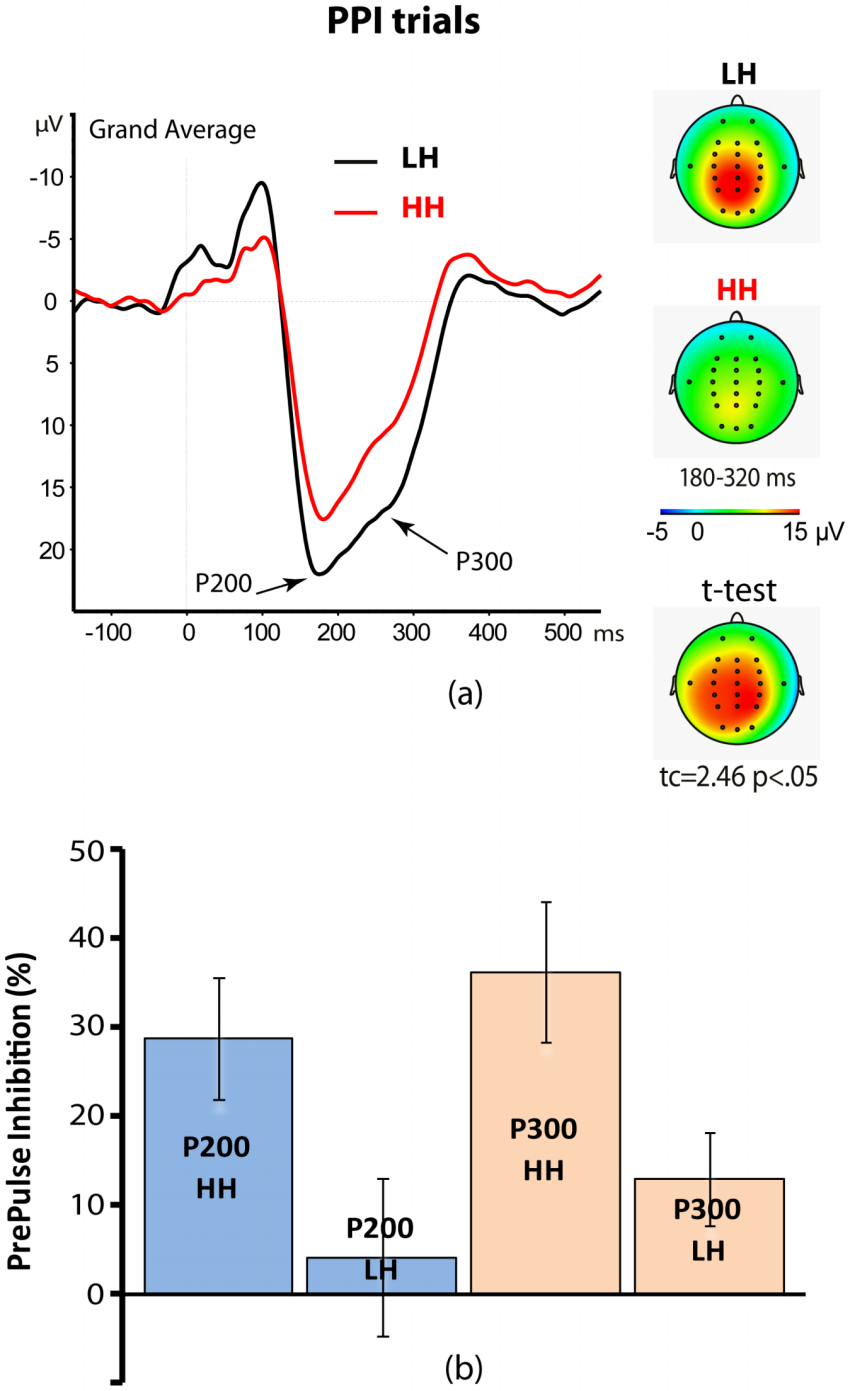
Grand average of ERP responses to auditory pulse of prepulse inhibition (PPI) trials (panel a, left side). Averaged scalp topography within a 180–320 msec time window including P200 and P300 waves (panel a, right-side). t-Test map of high (HH) vs. low (LH) hypnotizable participants is shown in the lower right side (panel a). Histogram shows the PPI of P200 and P300 amplitudes in HH and LH participants (panel b).

The ANOVA on PPI scores of the P200 and P300 amplitudes showed a main effect for hypnotizability for both ERP waves [P200: F(1,28) = 4.76, p = .037; P300: F(1,28) = 6.03, p = .020]. This effect demonstrated that the HH group had a significantly higher inhibition of the P200 and P300 waves than did the LH group (P200: 28.8±6.9% vs. 4.1±8.9%; P300: 36.24%±7.9% vs. 12.9%±5.2%, respectively for HH vs. LH group; see Figure 3a,b). PPI scores for the P200 wave yielded a significant effect for Scalp site [F(2,56) = 6.02, p = .012] that indicated a lower inhibition of P200 wave in the left side as compared to both the midline and right sides of the scalp (5.2±8.6% vs. 16.8±5.4%, and 18.3±7.3%, respectively). Moreover, for this wave, the interaction between Head level and PPI was significant [F(4,112) = 4.97, p = .0067]. This effect indicated that PPI effect on the midline P200 wave was significantly higher for PPI-60 and PPI-120 as compared to PPI-30 (27.3±6.0%, 19.9±6.8%, 3.3±3.5%, respectively; for PPI-60 vs. PPI-30: t = 3.75, p<.01; for 120 msec vs. 30 msec: t = 2.98, p<.05). For left and right sides of the scalp, the differences in P200 amplitudes among interstimulus intervals (ISIs) were not significant (left side: −8.2±10.5, 19.5±5.7, and 4.4±9.0; right side: 14.4±7.9%, 23.2±8.1%, and 17.2±8.5%; respectively for 30 msec, 60 msec, and 120 msec intervals; 0.4<*t*<1.5). Finally, PPI scores for the P300 wave evidenced a main effect for PPI [F(2,56) = 4.24, p = .020] that disclosed a smaller inhibition of the P300 wave for PPI at 30 msec as compared to 60 msec and 120 msec ISIs (10.1±4.3% vs. 25.8±7.0% and 24.0±5.7% t(29) = −2.98, and t(29) = −2.39, p<.05, respectively). No other main or interaction effects were observed for P200 and P300 wave.

### LORETA source localizations and individual differences in hypnotizability

**Figure 4:**
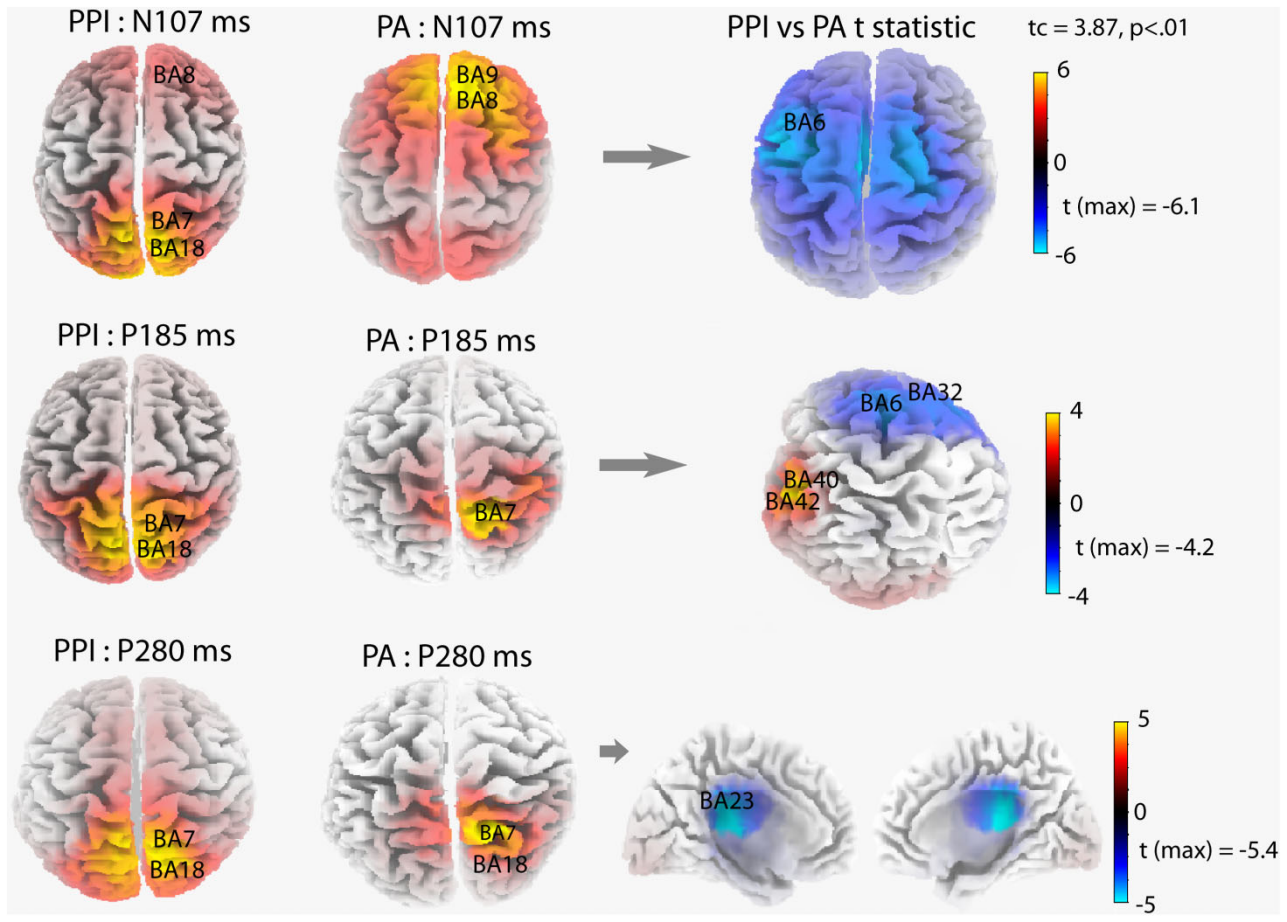
Source localization computed with sLORETA for N100 (107 msec), P200 (185 msec), and P300 (280 msec) components of the ERPs to prepulse inhibition (PPI) trials (left maps) and pulse-alone (PA) trials (middle maps). t-Test maps of PPI vs PA trials are reported in the right panel. Brodmann areas of maximal sLORETA values for ERP waves to PPI and PA trials and their significant differences are reported on each 3D map.

Figure 4 maps sLORETA solutions (i.e., z-current density at cortical voxels) modeling the distributed ERP sources of N100 and P200 and P300 peaks, respectively at 107, 185, and 280 msec.

MNI coordinates of maximal sLORETA values for the N100 wave to PA stimuli were estimated in the frontal lobe of the right hemisphere. Maximal values were in the right superior frontal gyrus (BA9: x = 10, y = 55, z = 40; BA8: x = 10, y = 50, z = 45), and middle frontal gyrus (BA9: x = 25, y = 50, z = 40; BA8: x = 5, y = 50, z = 45). Sources for both P200 and P300 waves to PA stimuli were estimated in the right parietal lobe. For the P200, sLORETA sources were estimated mainly in the right superior parietal lobule (BA7: x = 15, y = −65, z = 65), and right precuneus (BA7: x = 10, y = −65, z = 65). For the P300, sources were estimated mainly in the right superior parietal lobule (BA7: x = 20, y = −75, z = 55), and right precuneus (BA7: x = 10, y = −80, z = 50). 3D maps in the middle column of Figure 4 display the sources of N100, P200, and P300 waves, elicited by PA stimuli.

To estimate sources of N100, P200, and P300 waves of PP trials, sLoreta waveforms were averaged across the three prepulse-pulse trials (PPI-30, PPI-60, and PPI-120 msec). Common to all N100, P200, and P300 ERP waves were sources estimated in the bilateral precuneus (BA7: x = 5, y = −65, z = 65) and superior parietal lobule (BA7: x = 15, y = −65, z = 65; x = −15, y = −65, z = 65). 3D maps in the left column of Figure 4 display sources of N100, P200, and P300 waves, elicited by pulses of the PP trials.

To detect differences in regional brain activations between PP trials and PA trials, separate t-tests were performed for N100, P200 and P300 waves. For all ERP components, there were reduced activities to pulses of PP trials as compared to pulses of PA trials. The maximal significant activity reduction for the N100 (107 msec) and P200 (185 msec) waves occurred at BA6 in the left middle frontal gyrus (N100: x = −45, y = 5, z = 50; t = 6.13, p<.01; P200: x = −10, y = 5, z = 55; t = 4.19, p<.01). For the P200 wave, another source of significant activity reduction was found bilaterally at BA32 in the anterior cingulate gyrus (x = −5, y = 5, z = 50; t = 4.0, p<.01; x = 5, y = 5, z = 50; t = 3.8, p<.05). In addition, for the P200 there was significantly enhanced activity during PP trials at BA40 in the left postcentral gyrus (x = −65, y = −20, z = 15; t = 3.70, p<.05), and at BA42 in the left transverse temporal gyrus (x = −65, y = −20, z = 10; t = 3.58, p<.05). For the P300, the maximal activity reduction occurred bilaterally at BA23 in the posterior cingulate cortex (x = 5, y = −30, z = 25; t = 5.40, p<.01, and x = −5, y = −30, z = 25; t = 5.30, p<.01). Maps in the right column of Figure 4 display sources of these significant differences.

**Figure 5:**
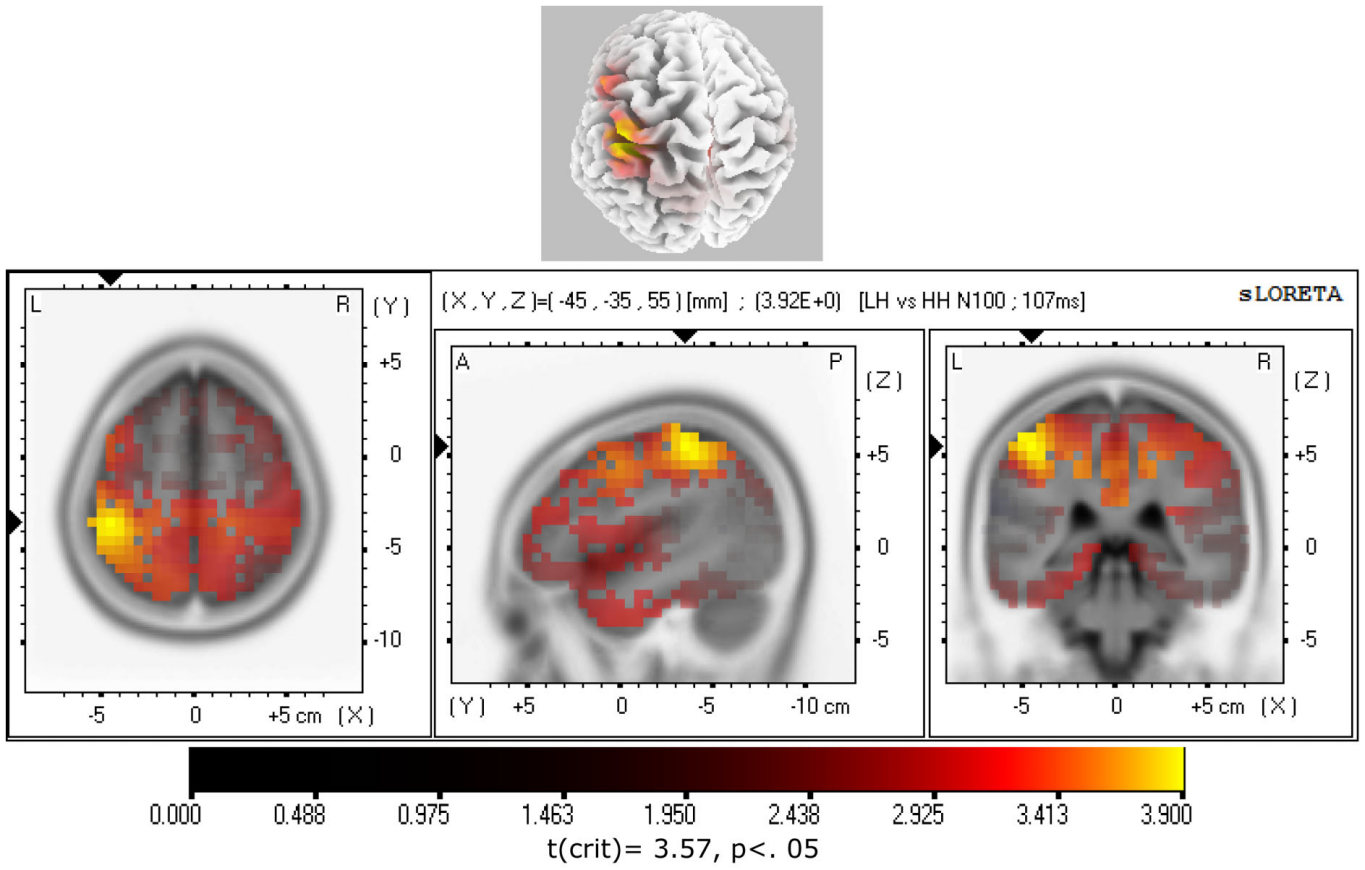
sLORETA parametric maps comparing N100 waves to pulse-alone (PA) trials of low (LH) vs. high (HH) hypnotizable participants. Note that HHs showed a reduced activation in the left postcentral gyrus (BA2) and inferior parietal lobule (BA40) at a time frame of 107 msec.

To test for statistically significant differences in regional brain activation between hypnotizability groups, the subtraction of ERP traces between hypnotizability groups were separately assessed using sLORETA source reconstruction for the N100, P200, and P300 (80–130 msec, 140–230 msec, and 250–300 msec time intervals, respectively). Separate t-tests between hypnotizability groups were performed for ERP waves elicited by PA and the pulse of prepulse-pulse trials. The only significant differences between hypnotizability groups was that the LH, as compared to HH, N100 wave to PA stimulus, showed a significant higher activity for the 107 msec time-frame in the left parietal lobe, mainly in the left postcentral gyrus (BA2) and inferior parietal lobule (BA40). These regions are reported in Table 2 and mapped in Figure 5.

**Table 2:** MNI coordinates and Brodmann areas (BA) of statistically stronger cerebral activation in low hynotizable (N = 15) as compared to high hypnotizable participants (N = 15) for N100 wave (107 ms) elicited by pulse-alone startle.

X(MNI)	Y(MNI)	Z(MNI)	t*	BA	Parietal Lobe
−45	−35	55	3.92	2	Postcentral Gyrus
−50	−35	60	3.86	40	Inferior Parietal Lobule
−40	−40	50	3.84	40	Inferior Parietal Lobule
−45	−40	50	3.84	40	Inferior Parietal Lobule
−45	−35	60	3.82	2	Postcentral Gyrus
−45	−40	55	3.81	40	Inferior Parietal Lobule
−50	−35	55	3.75	40	Postcentral Gyrus
−50	−40	55	3.75	40	Inferior Parietal Lobule
−40	−35	50	3.75	40	Inferior Parietal Lobule
−40	−40	55	3.74	40	Inferior Parietal Lobule
−40	−35	55	3.73	40	Postcentral Gyrus
−45	−35	50	3.72	40	Inferior Parietal Lobule
−40	−40	45	3.65	40	Inferior Parietal Lobule
−40	−45	50	3.65	40	Inferior Parietal Lobule
−45	−40	60	3.62	40	Inferior Parietal Lobule
−45	−45	50	3.62	40	Inferior Parietal Lobule

* t-crit. = 3.57, p<.05.

**Figure 6:**
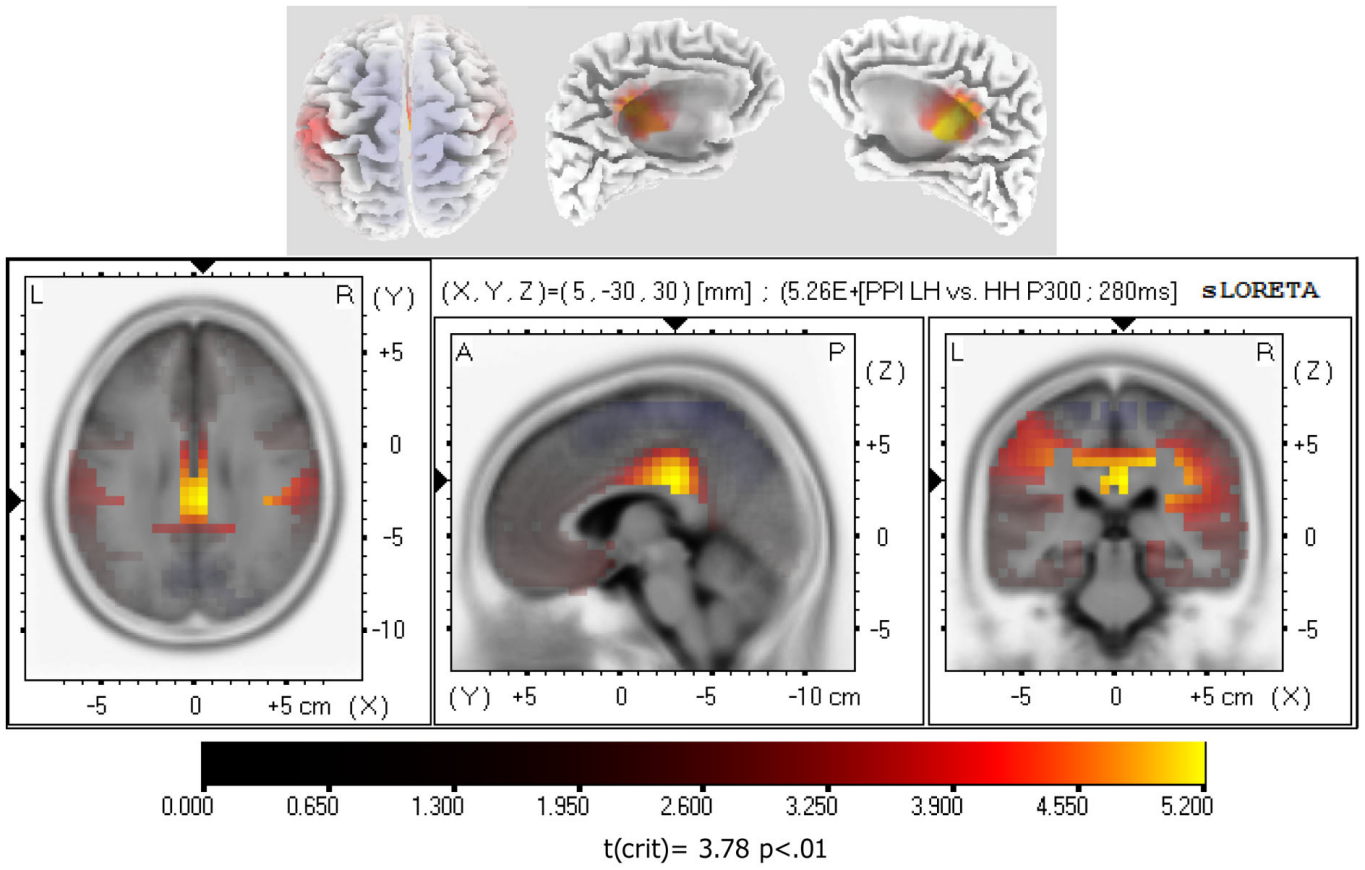
sLORETA parametric maps comparing P300 waves to prepulse inhibition (PPI) trials of low (LH) vs. high (HH) hypnotizable participants. Note that HHs showed a reduced activity mainly in the right, and to a less extent, in the left cingulate and posterior cingulate gyrus (BA23 and BA31) at a time frame of 280 msec.

Further, for PP trials, the HH group showed a significantly greater inhibition of the P300 wave than did the LH group, at a time-frame of 280 msec, in the limbic lobe, mainly in the right, and to a lesser extent, in the left cingulate and posterior cingulate gyrus (BA23 and BA31; see Table 3 and Figure 6). Comparisons by t-test between eyes-open hypnosis and waking condition in all participants, and by considering hypnotizability groups, did not yield any significant effects.

**Table 3:** MNI coordinates and Brodmann areas (BA) of statistically stronger cerebral inhibition in high hynotizable (N = 15) as compared to low hypnotizable participants (N = 15) for P300 wave (280 ms) during PP trials.

X(MNI)	Y(MNI)	Z(MNI)	t*	BA	Limbic Lobe
5	−30	30	5.26	23	Cingulate Gyrus
5	−30	25	5.11	23	Posterior Cingulate
0	−30	30	5.10	23	Cingulate Gyrus
5	−30	35	5.09	23	Cingulate Gyrus
0	−25	30	5.01	23	Cingulate Gyrus
20	−30	40	4.95	31	Cingulate Gyrus
−5	−30	30	4.92	23	Cingulate Gyrus
15	−30	40	4.86	31	Cingulate Gyrus
5	−20	30	4.84	23	Cingulate Gyrus
5	−35	25	4.80	23	Posterior Cingulate
20	−35	40	4.74	31	Cingulate Gyrus
10	−30	40	4.73	31	Cingulate Gyrus
0	−35	35	4.73	31	Cingulate Gyrus
15	−25	40	4.73	31	Cingulate Gyrus
0	−20	30	4.71	23	Cingulate Gyrus
−5	−30	25	4.62	23	Posterior Cingulate
5	−30	40	4.62	31	Cingulate Gyrus
15	−35	40	4.62	31	Cingulate Gyrus
5	−20	35	4.61	23	Cingulate Gyrus
0	−35	25	4.58	23	Cingulate Gyrus
0	−30	40	4.55	31	Cingulate Gyrus
−5	−20	30	4.54	23	Cingulate Gyrus
−10	−30	40	4.53	31	Cingulate Gyrus
−5	−30	40	4.53	31	Cingulate Gyrus
−15	−30	40	4.52	31	Cingulate Gyrus

* t-crit. = 3.88, p<.01.

## Discussion

### Startle blink and ERP responses to PA stimuli

Findings of the present study showed that PA stimuli elicited longer-latency EMG-startle responses and smaller frontocentral N100 amplitudes in HH compared to LH participants (Figure 2a). Moreover, the N100 wave was smaller in eyes-open hypnosis compared to the waking condition across all participants (Figure 2b). These findings parallel previous clinical reports of reduced N100 amplitude in bipolar/impulsive patients obtained using a paired-click paradigm [88]–[90], and reduced N100 and P200 in schizophrenics [91]. However, in the present study, we exclude the likelihood that individual differences between hypnotizability groups may be due to individual differences in fear and anxiety traits, since such differences in anxiety and fear scores were not significant (Table 1). We think that differences in attentional resources allocated for stimulus processing may account for differences between the hypnotizability groups. More specifically, HHs could allocate fewer attentional resources available to process fear-inducing stimuli – in this instance the PA – with the result that HH levels antagonize facilitatory effects of the fear system activated by these stimuli. However, considering that during eyes-open hypnosis, both hypnotizability groups had significant N100 wave reductions compared to a waking condition, we think that this N100 wave effect can be the product of both the hypnotic induction procedure per-se, and the suggested eyes-open relaxation state during hypnosis.

On the whole, the present findings confirm our earlier observations obtained using somatosensory painful stimulations that disclosed a reduced somatosensory N140 wave during hypnosis [92]. This finding also parallels previous observations of attenuated sound-elicited frontal N100 wave during hypnosis [93]. Finally, the present observation has provided experimental support to the hypothesis that hypnotizability and relaxation hypnosis may reduce attentional resources to process fear-inducing stimuli in the early stages of stimulus processing [94].

### PPI of the startle blink and ERP waves

We found higher PPI of the startle blink scores in HHs as compared to LHs, and we detected a greater PPI score at 120 msec interval as compared to PPI at 60 and 30 msec lead intervals in HHs, while we failed to find PPI differences across PPI intervals in LH participants. In terms of PPI of N100, P200, and P300 waves, we detected an increased PPI of these ERP components at lead intervals of 60 and 120 msec as compared to 30 msec. Both these PPI findings largely support the view that a central preattentive and protective mechanism facilitates the processing of a low-intensity stimulus in the face of potentially disruptive impact of a highly intense stimulus [30] and that a putative sensory-gating mechanism reduces ERP components [23].

The present PPI findings are in the opposite direction to those previously reported by Lichtenberg and colleagues [37], which obtained a reduced eyeblink PPI in HHs, suggesting dysfunctional sensorimotor gating in these individuals. Since we observed PPI differences between hypnotizability groups that were in the same direction for both eyeblink and ERP measures, we exclude the possibility that our results may be due to accidental artifacts. Moreover, in the present study we used a method to induce PPI that was similar to that used by the above mentioned authors [37], and thus we exclude the possibility that the opposite findings between the two studies can be due to differences in PPI testing. However, in order to account for the opposite PPI vs. hypnotizability trend between the two studies, we want to outline some differences that may account for differences in PPI responses. First, we measured eyeblink activity from the left side of the face, while Lichtenberg and colleagues, in line with previous studies [68], [95], measured blink activity from the right orbicularis oculi. Second, the present findings were obtained from a sample of women, while in the original study women and men were enrolled [37]. Gender may be a potential factor influencing the hypnotizability versus PPI relationship. This is because women have been found more susceptible to hypnosis than men [57], [58], although gender difference seems to be rather small when found [59], and because men are sometimes reported as showing smaller ASRs and a stronger PPI effect than women [62], [63]. Time of day is another possible confounding factor that was not controlled among previous PPI-hypnotizability studies. Research has demonstrated that time of day has an effect upon hypnotizability, with peaks at late afternoon and early evening [96]. An important difference between our and original studies [37], [40] lays in the fact that we excluded subjects with history of neurological or psychiatric diseases on the basis of a self-report questionnaire, while in previous studies a more reliable semi-structured interview was conducted by a psychiatrist with more than 10 years of clinical and research experience. Since a self-report questionnaire is not a very reliable tool for the exclusion of subjects, this clearly represents a limitation to the generalization of our findings. This methodological difference could account for contrasting findings between our and previous studies and it is a factor that should be controlled in future studies. Finally, another limitation of the present study, although common to previous reports, lays in the fact that we treated the HH participants as a homogeneous group and used aggregate stats, yet there is reason to assume that there are at least two different HH groups which may differ in PPI of the startle response. This assumption derived from recent findings indicating that highly hypnotizable individuals are distributed across two classes of response patterns, one suggesting an inward attention subtype and the other a dissociative subtype [97]. We applied Shapiro-Wilk' test on PPI startle responses in an attempt to test if the HH were normally distributed. This test did not disclose violations of the assumptions of homogeneity of variance within HH group for PPI scores, although in the HH group, for eyes-open hypnosis, the rejection of the null hypothesis of normality was near the significance level (W = 0.883, p = 0.053). Thus, we cannot exclude that existing differential response patterns in HH individuals may account for the apparent contrasting findings between our and previous findings [37]. There is thus clearly a need for further research controlling for potential heterogeneity of responses in HH individuals. Future research should explore the influence of the above mentioned eye-asymmetry factor, gender and time of day as potential mediators influencing the association of hypnotizability with PPI.

On the whole, our findings appear to support the neuropsychophysiological model proposed by Crawford and colleagues [1]–[4], [12] that HHs, in waking as well as in state of eyes-open hypnosis, have a more effective frontal attentional system implementing control, monitoring performance, and inhibiting unwanted stimuli from conscious awareness. In agreement with this view are previous observations reported by [41], who suggested a higher sensory gating efficiency in HHs. These authors reported that an increased anterior corpus callosum size was positively associated with hypnotizability and the ability to control pain. The rostral region of the corpus callosum, in conjunction with frontal cortex, plays a crucial role in attentional deployment and inhibitory control [98], [99], and influences the efficiency of the frontal cortices in sensory gating [100].

### Source localization findings

sLORETA analyses of the ERP waves of interest, elicited by PAs and pulses of prepulse-pulse trials, has identified core sources of activity (Figure 4). For the N100 wave of PA trials, these consisted of right superior and middle frontal gyri (BA8, BA9), while common to P200 and P300 waves were the sources in the right superior parietal lobule and precuneus (BA7, see left-side of Figure 4). For N100, P200, and P300 waves of PP trials, sources were estimated mainly in the bilateral precuneus, cuneus, and to a less extent, superior frontal gyrus (BA7, BA18 and BA8).

With regard to PPI as compared with the PA trials, for the N100 and P200 waves, the most pronounced reduction of activity was seen in the left middle frontal gyrus at BA6. This comparison for the P200 wave yielded another source of significant activity reduction bilaterally in the anterior cingulate gyrus at BA32, and two sources of enhanced activity in the left postcentral gyrus at BA40, and in the left transverse temporal gyrus at BA42. For the P300, a reduced activity occurred bilaterally at BA23 and BA31, in the posterior cingulate and cingulate cortices.

These findings parallel our previous findings [45] and indicate that bilateral frontal and, largely, parieto-occipital cortex seem to play an important role in the modulation of the startle responses (Figure 4). These findings appears in line with fMRI findings that lower PPI is associated with reduced activity in the inferior frontal gyrus, insula extending to putamen, thalamus, parahippocampal gyrus, inferior parietal and middle temporal regions [101], and in the orbitofrontal cortex [102], [103].

The present study, for the N100 (107 msec) elicited during PA trials, has evidenced in HHs compared to LHs, a reduced activation in the left parietal lobe, mainly in the left postcentral gyrus (BA2) and inferior parietal lobule (BA40; Table 2 and Figure 5). Since N100 has been suggested as a reliable measure of sensory gating [91], [104], these findings suggest a more efficient sensory gating in HHs, compared with LHs, and indicate that left parietal lobe is of importance. Moreover, in HHs, auditory pulses during PP trials yielded a reduced activity for the P300 at 280 msec, in the cingulate and posterior cingulate gyrus (BA23 and BA31, see Table 3 and Figure 6). The PCC is involved in visuospatial orientation and assessment of self-relevance of emotional events and stimuli [43], [105]. The activation of PCC is an important component in the preparation for coping with a physical threat [106]. On this basis, it is reasonable to conclude that the reduced activity observed in left-parietal lobe, and enhanced inhibition in posterior cingulate cortex, may reflect the reduced sensitivity (or enhanced avoidance) of HHs to startle-negative stimuli.

Finally, although sLORETA provides a good localization accuracy, a major limitation of the present study is in the fact that only 22 electrodes were used for source analysis. This reduces the spatial resolution, and with impaired spatial resolution, there is a smaller chance that sLORETA will be able to separate two closely spaced sources [107], [108]. Thus, a greater number of recording electrodes is recommended in future studies to enhance spatial resolution. Further studies are necessary to replicate our findings and to investigate the issues raised here.

Another limitation of the present study lies in the fact that our findings are restricted to women participants and, thus, cannot be generalized to men. Further studies would do well to consider gender, time of day, eye-recording asymmetry factors, and tested heterogeneity of HH individuals as potential mediators of the association between hypnotizability, hypnosis, and PPI.

The present work has demonstrated a significantly higher PPI of eyeblink startle (Figure 1) and higher inhibition of N100 wave to PA stimuli in the left parietal lobe (BA2/40; Figure 2a, Figure 5) in HHs compared to LHs. In addition, in the former hypnotizability group compared to the latter, we observed a higher PPI of the P200 wave (Figure 3a,b) and of the P300 wave in the cingulate and posterior cingulate gyrus (BA23/31; Figure 3b and Figure 6). Further, eyes-open hypnosis, compared to a waking control condition, yielded a smaller N100 wave to PA stimuli (Figure 2b) and a greater PPI of this component. With regard to individual differences on eyeblink startle, the present PPI-startle findings appear in the opposite direction to those reported in previous studies [37], [40] wherein it was thought to suggest a dysfunctional sensorimotor gating in HH individuals. In contrast, overall findings of the present study indicate a more efficient sensory gating in HHs, compared with LHs and that HHs could allocate fewer attentional resources available to process startle eliciting stimuli – with the result that higher hypnotizability levels antagonize facilitatory effects of the fear system activated by these stimuli. To disentangle the apparent contrasting findings between our and previous studies [37], [40], further research is needed, devoted to control a number of previously uncontrolled factors, i.e., the side of measured eye-blink activity, gender, time of day, and heterogeneity of the startle response within the HH group [97].
